# Study on the Application of Machine Learning of Melt Pool Geometries in Silicon Steel Fabricated by Powder Bed Fusion

**DOI:** 10.3390/ma19010068

**Published:** 2025-12-24

**Authors:** Ho Sung Jang, Sujeong Kim, Jong Bae Jeon, Donghwi Kim, Yoon Suk Choi, Sunmi Shin

**Affiliations:** 1 Smart Forming Process Group, Korea Institute of Industrial Technology, Ulsan 44776, Republic of Korea; ggi0505@kitech.re.kr; 2 School of Materials Science and Engineering, Pusan National University, Busan 46241, Republic of Korea; sujeong0530@pusan.ac.kr; 3 Department of Materials Science and Engineering, Dong-A University, Busan 49315, Republic of Korea; jbjeon@dau.ac.kr; 4 Computational Materials Science and Engineering Team, Hyundai Motor Group, Uiwang 16082, Republic of Korea; donghwi.kim@hyundai.com

**Keywords:** selective laser melting, silicon steel, additive manufacturing, process optimization, machine learning

## Abstract

In this study, regression-based machine learning models were developed to predict the melt pool width and depth formed during the Laser Powder Bed Fusion (LPBF) process for Fe-3.4Si and Fe-6Si alloys. Based on experimentally obtained melt pool width and depth data, a total of 11 regression models were trained and evaluated, and hyperparameters were optimized via Bayesian optimization. Key process parameters were identified through data preprocessing and feature engineering, and SHAP analysis confirmed that the input energy had the strongest influence on both melt pool width and depth. The comparison of prediction performance revealed that the support vector regressor with a linear kernel (SVR_lin) exhibited the best performance for predicting melt pool width, while the multilayer perceptron (MLP) model achieved the best results for predicting melt pool depth. Based on these trained models, a power–velocity (P-V) process map was constructed, incorporating boundary conditions such as the overlap ratio and the melt pool morphology. The optimal input energy range was derived as 0.45 to 0.60 J/mm, ensuring stable melt pool formation. Specimens manufactured under the derived conditions were analyzed using 3D X-ray CT, revealing porosity levels ranging from 0.29% to 2.89%. In particular, the lowest porosity was observed under conduction mode conditions when the melt pool depth was approximately 1.0 to 1.5 times the layer thickness. Conversely, porosity tended to increase in the transition mode and lack of fusion regions, consistent with the model predictions. Therefore, this study demonstrated that a machine learning-based regression model can reliably predict melt pool characteristics in the LPBF process of Fe-Si alloys, contributing to the development of process maps and optimization strategies.

## 1. Introduction

Silicon steel is a representative soft magnetic alloy, typically containing approximately 3 wt.% Si [1]. As the Si content increases, core loss decreases and magnetic performance improves. However, exceeding 4 wt.% Si leads to an increase in brittleness, thus posing difficulties for rolling [2]. Therefore, it is necessary to develop an efficient manufacturing method that overcomes the limitations of existing processes and offers high design flexibility. Recently, additive manufacturing (AM) technology has attracted attention for its ability to realize complex shapes, reduce weight, enhance efficiency and design flexibility. Among various AM processes, Laser Powder Bed Fusion (LPBF) has emerged as a promising technique due to its capability to locally melt and consolidate metal powders in a layer-by-layer manner [3,4,5].

In the LPBF process, interactions between the laser and powder occur over millisecond timescales and micrometer length scales. This process leads to complex phenomena such as rapid solidification, non-equilibrium phase transitions, and vaporization phenomena [6,7,8]. In particular, the melt pool width and depth affect the morphological characteristics of the melt pool and directly influence final product properties such as density, mechanical properties, and magnetic properties. Melt pool formation can be categorized into conduction mode and keyhole mode. The conduction mode is comparatively stable but susceptible to cause lack of fusion defects [9]. The keyhole mode offers deep penetration and high energy efficiency but introduces instability and porosity formation [10].

Previous research on LPBF of Fe–Si alloys has primarily focused on microstructure and magnetic properties, with relatively few studies addressing the systematic prediction and control of melt pool geometry. In process development and optimization for new materials, traditional approaches have relied heavily on experimental design or physics-based simulations such as finite element modeling (FEM) and computational fluid dynamics (CFD). While such methods are valuable, they are also limited by high computational cost and extensive trial-and-error [11,12,13]. Furthermore, physics-based simulation approaches have been predominantly applied to elucidate fundamental mechanisms—such as keyhole formation, microstructural evolution, and defect generation—rather than to systematic process optimization.

In this context, machine learning (ML) is gaining attention as an alternative approach capable of capturing complex, nonlinear relationships between process parameters and melt pool behavior. ML has been successfully applied to various aspects of LPBF, including property prediction, defect detection, and real-time monitoring [14,15,16,17]. Scime et al. [18] used ML to predict keyhole defects and balling instability in the LPBF process of Inconel 718 alloy. They suggested the potential of using machine learning models to create process maps. Yuan et al. [19] developed a predictive model for single-track defects using machine learning-based monitoring. Real-time evaluation of LPBF data successfully led to the formation of a proper single track, providing on-site quality detection and real-time monitoring. G Tapia et al. [20] established a Gaussian process (GP) model based on single-track experimental melt pool depth and process parameters, including laser power, scan speed, and laser beam size. The derived normalized enthalpy criterion and process map provided evidence that the GP model can successfully predict melt pool depth. These studies suggest strong applicability of ML to Fe–Si LPBF as well.

This study aimed to predict the molten pool width and depth formed during the LPBF process for Fe-3.4Si and Fe-6Si alloys, and to derive optimal process conditions based on these predictions. A machine learning regression model was constructed based on experimental data, and the contribution of key factors was assessed through SHAP analysis. Furthermore, the predicted results under both training and unseen conditions were used to create a P-V process map with boundary conditions. The reliability of the model and the potential for process optimization were verified by 3D X-ray CT through porosity analysis of samples manufactured under the derived conditions.

## 2. Materials and Methods

### 2.1. Framework and Data Preparation

**Figure 1:**
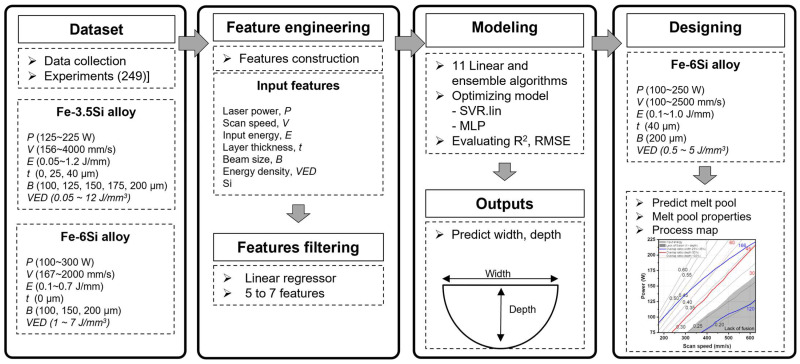
Schematic diagram of the proposed approach for process parameter optimization.

In this study, a machine learning framework was developed to predict the melt pool width and depth based on experimental datasets for Fe-3.4%Si and Fe-6%Si alloys. Data for the Fe-3.4%Si alloy were obtained at layer thicknesses of 0, 25, and 40 μm, while data for the Fe-6%Si alloy were collected at a layer thickness of 0 μm. The data at 0 μm correspond to single-track experiments on a substrate without a powder layer. The substrate was fabricated using the same alloy composition as the powder to minimize material property discrepancies. Although this represents a physically distinct regime from powder bed fusion, it was encoded as a continuous variable to allow the model to learn the trend of energy demand. Prior to model training, outliers were rigorously identified to ensure data quality. Samples with physical defects (e.g., balling) preventing measurement were excluded. As a result, 27 samples were removed from the initial dataset, yielding a final dataset of 249 samples for model training. The trained models were then applied to predict the melt pool geometry of Fe–6Si at a layer thickness of 40 μm, followed by defect analysis of the fabricated specimens. Figure 1 schematically illustrates the prediction approach proposed in this study.

A total of 11 machine learning models based on scikit-learn were used: Linear Regression (LR), Ridge Regression (Rd), Support Vector Regressor with a radial basis function kernel (SVR_rbf) and a linear kernel (SVR_lin), K-Nearest Neighbor (KNN), Decision Tree Regressor (DT), Random Forest (RF) Regressor, Gradient Boost Regressor (GB), Extreme Gradient Boost Regressor (XGB), Adaptive Boost (AdaBoost) Regressor (ADA), and Artificial Neural Network (Multi-layer Perceptron, MLP). Each model was trained independently to predict the melt pool width and depth. Hyperparameters were optimized via Bayesian optimization, ensuring efficient convergence and avoiding overfitting.

**Figure 2:**
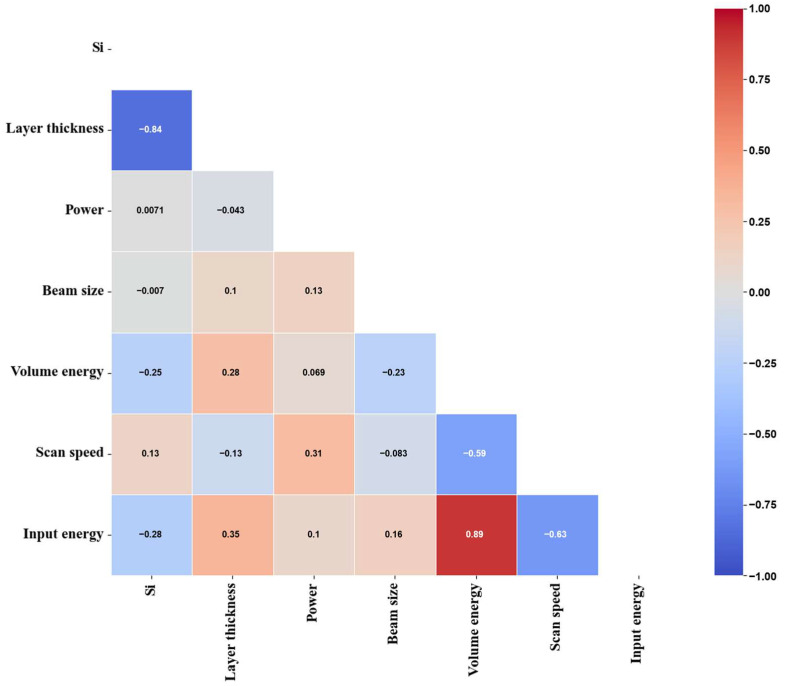
Features filtering by Pearson correlation coefficient of the initial feature candidates.

The input features included laser power, scan speed, input energy, volume energy density, layer thickness, beam size, and Si content. After removing missing entries and outliers, 249 valid samples remained. All input features were normalized using the MinMax scaler, while the target features retained their original scale. The optimal feature combination was derived through a feature engineering process. Pearson correlation coefficients (PCC) indicated no strongly collinear pairs (|r| > 0.9) among retained features (Figure 2). To find the optimal number of influential features, we performed forward inclusion based on feature importance and executed 5-fold cross-validation repeated 100 times. The optimal feature sets for width and depth were chosen by minimizing the root-mean-square error (RMSE). The dataset was split in a 4:1 ratio for training and evaluation. Each model was trained and evaluated 100 times on randomly resampled subsets. Model performance was evaluated using the coefficient of determination (R^2^). The best-performing models were subsequently used to construct the process maps.

### 2.2. Sample Fabrication and Characterization

The machine learning-derived process map was used to guide sample fabrication. The laser power was varied from 100 W to 300 W (typical step: 25 or 50 W, depending on the alloy), and the input energy was varied from 0.05 to 1.2 J/mm (step: 0.05 or 0.1 J/mm), resulting in a scan speed ranging from 156 mm/s to 1000 mm/s. The layer thickness was fixed at 40 μm, and the beam size at 200 μm. Laser power and scan speed were varied systematically to evaluate their effects on melt pool geometry and porosity. Fabrication was performed using an OPM-250L system (Sodick Co., Ltd., Schaumburg, IL, USA). Because hatch-spacing control is not available on this platform, hatch spacing was fixed at 80 µm. Processing was conducted under nitrogen atmosphere with oxygen levels maintained below 1%, and the substrate was held at 100 °C. Fe–6Si alloy powder was used, and 10 mm × 10 mm × 10 mm cubes were fabricated. The fabricated specimens were analyzed for internal defects and porosity using 3D X-ray computed tomography (240-kV X-ray, resolution—29 µm; XTH320, Nikon, Tring, UK).

## 3. Results and Discussion

### 3.1. Model Development

**Figure 3:**
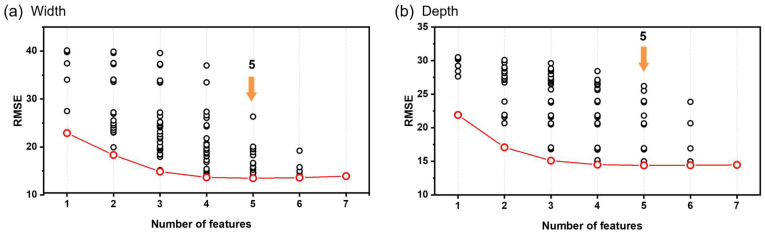
The best feature subset selection of the feature candidates. The red circles indicate the feature combinations with the lowest RMSE for each number of features.

To reduce model complexity and mitigate overfitting, we determined the optimal number of key features using a forward selection approach based on the LR model. As the number of features increased, the RMSE tended to decrease, but there was no significant decline after a certain interval (Figure 3). Therefore, candidate feature sets factors were selected based on the interval with the lowest RMSE and the most stable values. For melt pool width prediction, laser power, scan speed, input energy, layer thickness, and Si content were identified as key factors. For melt pool depth prediction, laser power, input energy, beam size, volume energy, and Si content were identified as the dominant parameters (Table 1). Notably, input energy, laser power, and Si content significantly affected both width and depth. In contrast, layer thickness and scan speed were more influential for width, whereas beam size and volumetric energy dominated depth prediction.

**Figure 4:**
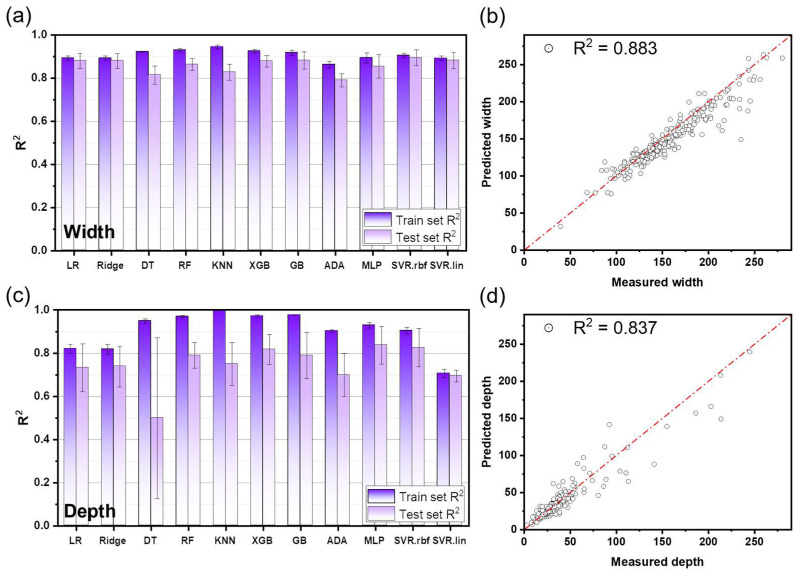
(**a**,**c**) Predictability comparison of eleven machine learning algorithms trained by datasets. Measured (**b**) width and (**d**) depth versus predicted width and depth plots.

The models were initially evaluated using default hyperparameters to provide a baseline comparison across different algorithms. While some models showed relatively higher baseline performance under default settings, the predictive performance and generalization behavior varied substantially depending on the model type. To systematically improve model performance and ensure a fair comparison, Bayesian hyperparameter optimization was applied to all eleven candidate models using the same cross-validation protocol. The resulting optimized models were then evaluated in terms of training and testing performance, as summarized in Table 2 and Figure 4. For melt pool width prediction, R^2^ values ranged from 0.863 to 0.945 for training and 0.824 to 0.881 for testing (Figure 4a). For melt pool depth prediction, R^2^ values ranged from 0.706 to 0.998 for training and 0.498 to 0.837 for testing (Figure 4c). In predicting melt pool width and depth, tree-based models exhibited a significant gap between training and testing performance, and a tendency toward overfitting. This phenomenon is consistent with problems commonly reported in small datasets [21]. Tree-based models have high expressive power, allowing them to construct very complex models. Furthermore, they can overfit due to noise in the training data [22,23].

**Figure 5:**
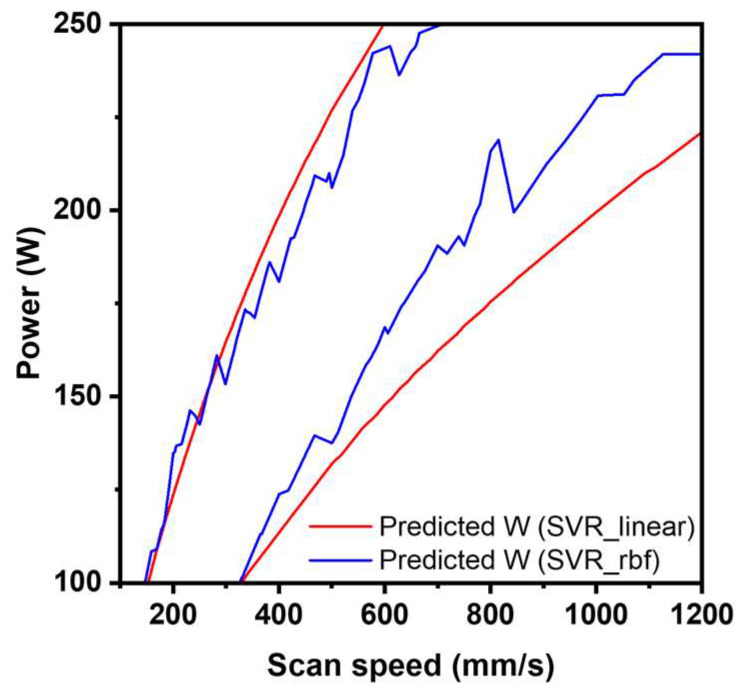
Comparison of SVR_linear and SVR_rbf for contour line for Predicted width (133 ≤ *w* ≤ 166 μm).

In the case of melt pool width prediction, the SVR_rbf achieved the highest performance with an R^2^ of 0.895, but showed signs of overfitting (Figure 4a,b). Therefore, the next best and more stable SVR_lin was selected as the final width predictor (Figure 5). The SVR_lin’s prediction performance on the training dataset was stable, with R^2^_train of 0.892 ± 0.011, R^2^_test of 0.882 ± 0.038, and R^2^_100-run mean test of 0.883. SVR is well suited to small-to-medium datasets and supports both linear and nonlinear regression [24,25,26]. In particular, it has been reported that RBF kernel-based SVR can easily overfit depending on hyperparameter settings [27].

For melt pool depth, The MLP model demonstrated the best generalization capability, with R^2^_train of 0.929 ± 0.012, R^2^_test of 0.837 ± 0.087, and R^2^_100-run mean test of 0.837 (Figure 4c,d). This is consistent with previous studies where the SVR and MLP were effectively used to predict melt pool characteristics and weld bead shape [28,29,30]. Accordingly, SVR_lin and MLP were adopted as the final models for width and depth prediction, respectively.

### 3.2. Feature Importance and SHAP Analysis

**Figure 6:**
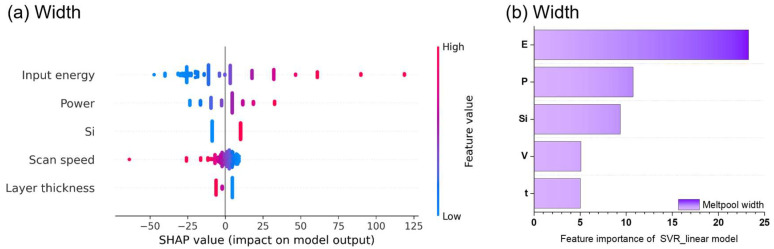
(**a**) SHAP values of the features and (**b**) feature importance by SVR_linear model for predict width.

**Figure 7:**
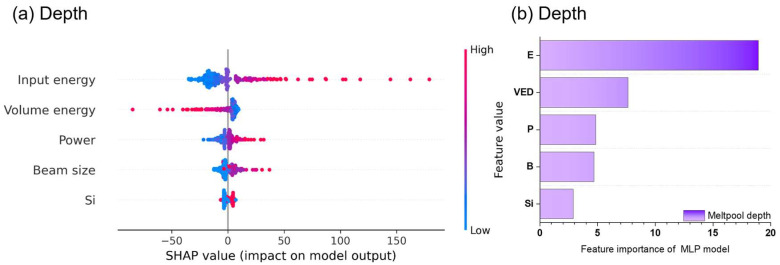
(**a**) SHAP values of the features and (**b**) feature importance by MLP model for predict depth.

To better interpret the trained models, Shapley Additive Explanations (SHAP) analysis was employed to identify the relative importance of input variables [31]. SHAP confirmed that input energy was the most influential feature for both melt pool width and depth (Figure 6 and Figure 7). Increasing laser power elevates the peak temperature at the melt pool surface, while decreasing scan speed extends the interaction time between the laser and the melt pool surface. This suggests that the size of the melt pool can be controlled by increasing or decreasing the input energy, consistent with previous studies [6,32].

For width prediction, the influences ranking was: input energy > laser power > Si content > volume energy > scan speed > layer thickness (Figure 6a,b). The melt pool width tended to increase with input energy. Increasing layer thickness required more input energy to form the melt pool, which tended to decrease the melt pool width. This finding is consistent with previous research showing that as the layer thickness increases, the melt pool width becomes relatively smaller, leading to a decrease in the melt pool width-to-depth ratio [32].

For depth prediction, the ranking was: input energy > volume energy > laser power > beam size > Si content (Figure 6a,b). Although higher volumetric energy generally leads to deeper melt pools [33,34], an interesting trend was observed: increasing beam diameter also correlated with deeper pools, even when volumetric energy density decreased. This behavior originates when an increase in laser beam size causes the surface temperature to remain below a critical transition threshold (*T_t_*). In this temperature regime, the sign of the surface-tension temperature coefficient (*dγ*/*dT*) can shift to favor an inward Marangoni flow, effectively reversing the typical outward flow and guiding the molten material toward the pool center. Consequently, this inward flow enhances downward heat transport and increases penetration depth, leading to deeper melt pools under the corresponding processing conditions [35,36].

### 3.3. Reverse Engineering and Process Map Derivation

**Figure 8:**
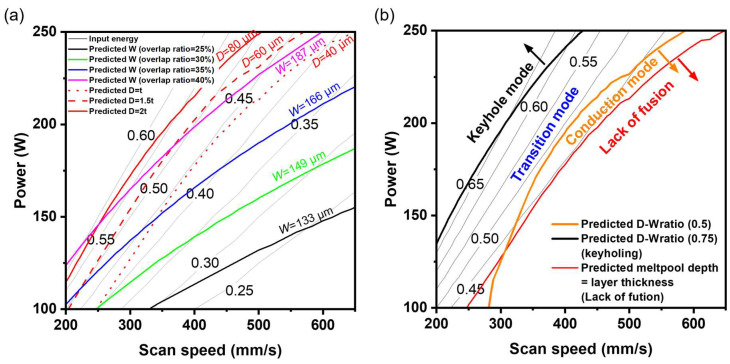
Process parameter map predicted by SVR_linear and MLP model with (**a**) overlap ratio and (**b**) melt pool mode. The gray lines represent input energy contours.

Based on the trained SVR_lin and MLP model, a power–velocity (P-V) process map with boundary conditions was established for Fe-6%Si alloy with a layer thickness of 40 μm (Figure 8). First, the overlap ratio criterion was considered as a boundary condition (Figure 8a). The melt pool width is recommended to be between 1.5 and 2.5 times the hatch spacing, which corresponds to a melt pool width of 120 μm to 200 μm [37,38,39]. An inappropriate overlap ratio can lead to defects such as porosity, lack of fusion, and balling due to insufficient powder melting [40,41]. Furthermore, excessive overlap ratios have been reported to elevate the local temperature, consequently increasing both the melt pool width and depth [42]. Based on a 25–40% overlap criterion, the melt pool width was defined with bounds of 133 to 187 μm, and the melt pool depth constrained to 1 to 2 times the layer thickness.

Second, the process conditions were constrained by the depth-to-width ratio (D-W ratio), which determines the melt pool morphology (Figure 8b). The modes were categorized as conduction (D-W ratio < 0.5), transition (0.5 ≤ D-W ratio < 0.75), and keyhole (D-W ratio ≥ 0.75) [39]. The lack of fusion (LOF) region was defined by a melt pool depth shallower than the layer thickness, consistent with prior reports [43]. Considering the boundary conditions, input energy of 0.45~0.60 J/mm was derived to avoid both the keyhole and lack of fusion (LOF) regions, with the power fixed at 180 W.

**Figure 9:**
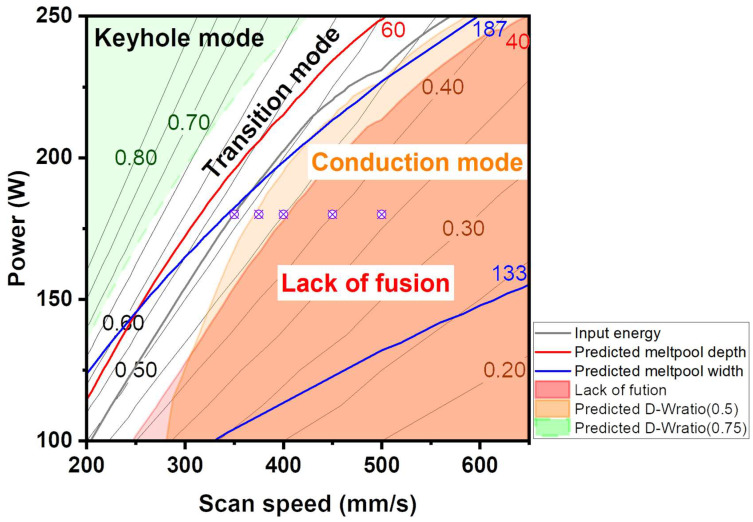
Process parameter map with different criteria (overlap ratio and melt pool morphology) predicted using the SVR_linear and MLP models. The gray lines represent input energy contours.

Based on these criteria of overlap ratio and melt pool morphology, an L-PBF process strategy suitable for Fe-6Si alloys was proposed (Figure 9). In the process map, the blue contour line enforces the melt pool width target (133 < W < 187 μm), and the red contour line enforces the melt pool depth target (40 < D < 80 μm). The shaded red region highlights conditions susceptible to Lack of Fusion (LOF) defects. Based on melt pool morphology characteristics, the remaining regions are classified as keyhole mode (light green area), conduction mode (orange area), with the area between them representing the transition mode. By fixing the laser power at 180 W and adjusting the scan speed from 350 to 500 mm/s, samples were fabricated across Input energy of approximately 0.36~0.51 J/mm to experimentally probe the recommended processing window.

### 3.4. Experimental Validation

**Figure 10:**
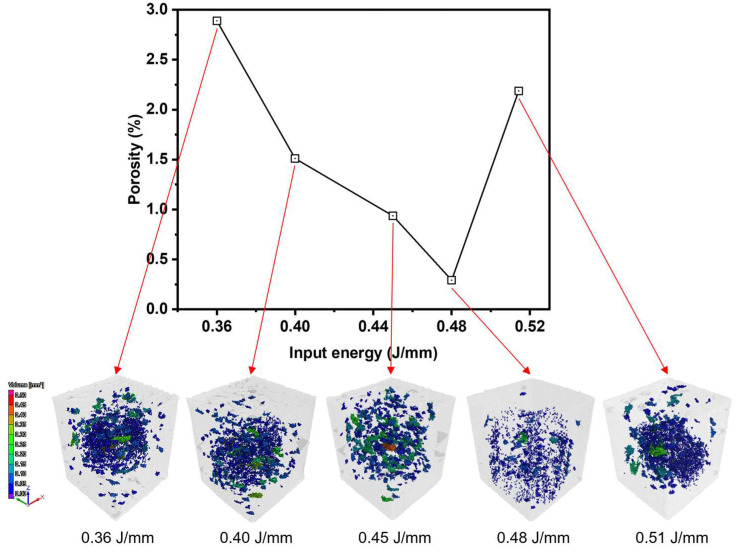
Porosity measurement results and internal defect visualization in a cubic sample using X-ray Computed Tomography.

The proposed processing window was validated through fabrication of cubic specimens and subsequent 3D X-ray computed tomography analysis (Figure 10). The measured porosity ranged from 0.29% to 2.89%, and the volume of individual pores was confirmed to be less than 0.5 mm^3^ across all builds. Overall, while higher input energy generally reduced porosity, the porosity increased again when the melt pool depth exceeded approximately 1.5 times the layer thickness and the melt pool morphology entered a transition mode. The trend of increasing porosity in keyhole mode was consistent with previous studies [44,45]. Furthermore, specimens fabricated in the LOF region exhibited over a porosity exceeding 0.94%. Conduction mode melt pools with depths ranging from 1.0 to 1.5 times the layer thickness exhibited the lowest porosity. The sample fabrication outcomes successfully validated the efficacy of the optimal region defined in the proposed process map. These results simultaneously highlighted the narrowness of the robust operating window, revealing that the stable region for process optimization is highly limited, and that there is a risk of low-quality samples due to defect formation. The narrow robust operating window observed in this study is a common characteristic of many non-weldable alloys. Recent studies have reported that Ultrasonic Vibration Assisted Laser Manufacturing (UVA-LM) can effectively expand this processing window by stabilizing melt pool dynamics [46]. This suggests that the ML-driven process optimization framework proposed in this study has broad applicability to materials exhibiting high crack sensitivity and restrictive process margins. This study proved the utility of the approach for process optimization by demonstrating that regression models can effectively guide the exploration of untrained data regions. Furthermore, integrating advanced computational methods, such as phase-field models applicable to AM and capable of providing deep insights into solidification mechanisms, is expected to provide even deeper insights [47,48]. Combining our ML-based process optimization with high-precision physics-based simulations offers a complementary perspective, which is expected to lead to a more comprehensive understanding of the process–structure–property relationship in future research.

## 4. Conclusions

This study developed regression-based machine learning models to predict the melt pool width and depth for Fe-3.4Si and Fe-6Si alloys processed by L-PBF. The melt pool width and depth of the Fe-6Si alloy were predicted not only from trained data but also from untrained regions, and a process map was derived based on this prediction.

Key process parameters significantly influencing melt pool geometry were identified through feature engineering and SHAP analysis, with input energy exhibiting the greatest impact. Among the evaluated models, the SVR_lin model showed superior performance for melt pool width prediction, while the MLP model achieved the best performance for depth prediction, with R^2^ values exceeding 0.83 in testing. Based on these models, a power–velocity (P-V) process map was derived, incorporating both overlap ratio and melt pool morphology constraints. The optimal processing window was identified at an input energy range of 0.45–0.60 J/mm under a fixed laser power of 180 W.

Experimental validation using 3D X-ray computed tomography confirmed that conduction-mode melt pools with depths approximately 1.0–1.5 times the layer thickness resulted in the lowest porosity (0.29%). In contrast, transition and keyhole regimes exhibited increased porosity, demonstrating the high reliability of the machine learning-based process map for identifying stable processing windows. Overall, this study highlights the effectiveness of machine learning in accurately predicting melt pool geometries and guiding process optimization in LPBF of Fe–Si alloys.

## Figures and Tables

**Table 1:** Feature candidates obtained from feature engineering.

Sample	Width	Depth
Best features	‘Power’, ‘Scan speed’, ‘Input energy’, ‘Layer thickness’, ‘Si’	‘Power’, ‘Input energy’, ‘Beam size’, ‘Volume energy’, ‘Si’

**Table 2:** Hyperparameters optimized for SVR_lin, MLP.

Target	ML Algorithm	Hyperparameters Optimized
Width	Support Vector Regression	C = 341, kernel = ‘linear’, epsilon = 11.610503225584356 (µm), tol = 0.5063349249279957
Depth	Multi-layer Perceptron regressor	Hidden1: 331, Hidden2: 121, alpha: 0.05861435807687141, learning rate: 0.03472671856325738, batch_size: 38, momentum: 0.18279174066800405, tol: 0.009048802539977611

## Data Availability

The original contributions presented in this study are included in the article. Further inquiries can be directed to the corresponding authors.
